# Freeze vs. Spray Drying for Dry Wild Thyme (*Thymus serpyllum* L.) Extract Formulations: The Impact of Gelatin as a Coating Material

**DOI:** 10.3390/molecules26133933

**Published:** 2021-06-28

**Authors:** Aleksandra A. Jovanović, Steva M. Lević, Vladimir B. Pavlović, Smilja B. Marković, Rada V. Pjanović, Verica B. Đorđević, Viktor Nedović, Branko M. Bugarski

**Affiliations:** 1 Faculty of Technology and Metallurgy, University of Belgrade, Karnegijeva 4, 11000 Belgrade, Serbia; rada@tmf.bg.ac.rs (R.V.P.); vmanojlovic@tmf.bg.ac.rs (V.B.Đ.); branko@tmf.bg.ac.rs (B.M.B.); 2 Faculty of Agriculture, University of Belgrade, Nemanjina 6, 11080 Belgrade, Serbia; slevic@agrif.bg.ac.rs (S.M.L.); vlaver@agrif.bg.ac.rs (V.B.P.); vnedovic@agrif.bg.ac.rs (V.N.); 3 Institute of Technical Sciences of SASA, Knez Mihailova 35/IV, 11000 Belgrade, Serbia; smilja.markovic@itn.sanu.ac.rs

**Keywords:** encapsulation, freeze drying, gelatin, polyphenols, spray drying

## Abstract

Freeze drying was compared with spray drying regarding feasibility to process wild thyme drugs in order to obtain dry formulations at laboratory scale starting from liquid extracts produced by different extraction methods: maceration and heat-, ultrasound-, and microwave-assisted extractions. Higher total powder yield (based on the dry weight prior to extraction) was achieved by freeze than spray drying and lower loss of total polyphenol content (TPC) and total flavonoid content (TFC) due to the drying process. Gelatin as a coating agent (5% *w*/*w*) provided better TPC recovery by 70% in case of lyophilization and higher total powder yield in case of spray drying by diminishing material deposition on the wall of the drying chamber. The resulting gelatin-free and gelatin-containing powders carried polyphenols in amount ~190 and 53–75 mg gallic acid equivalents GAE/g of powder, respectively. Microwave-assisted extract formulation was distinguished from the others by a higher content of polyphenols, proteins and sugars, higher bulk density and lower solubility. The type of the drying process mainly affected the position of the gelatin-derived -OH and amide bands in FTIR spectra. Spray-dried formulations compared to freeze-dried expressed higher thermal stability as confirmed by differential scanning calorimetry analysis and a higher diffusion coefficient; the last feature can be associated with the lower specific surface area of irregularly shaped freeze-dried particles (151–223 µm) compared to small microspheres (~8 µm) in spray-dried powder.

## 1. Introduction

*Thymus serpyllum* L. (wild thyme, Lamiaceae) synthesizes and stores numerous biochemical products: proteins, polysaccharides, fiber, essential oil, monoterpenes, and polyphenols [1,2]. Polyphenolic compounds have recently attracted increasing interest in the food, pharmaceutical and cosmetic industries due to their biological activities, such as antioxidant, antimicrobial, spasmolytic, anti-inflammatory, cardioprotective, anti-allergic, and anti-carcinogenic activities [3,4].

However, polyphenols, like many other bioactive compounds in liquid formulations, are generally susceptible to both chemical and physical degradation. Dry formulation obtained by either lyophilization (freeze drying) or spray drying is often the alternative. Lyophilization uses freezing and low pressure with the addition of heat (in the amount necessary to provide the sublimation of frozen water) to obtain dehydrated product [3]. It is known that lyophilized particles with polyphenols are stable over long periods due to the prevention of hydrolytic and oxidative degradation of the active compounds during storage [4]. However, freeze drying is an expensive and time-consuming process, about six times more expensive per kg of water removed in comparison with spray drying. Spray drying is an industrial technology which uses hot air or inert gas to obtain powder from a solution. The mentioned technique enhances the microbiological and biochemical stability of products, and provides a reduction in storage and transport costs [3]. Although biopharmaceuticals are generally more stable in the dried state, it is well known that lyophilization and spray drying processes themselves could modify the antioxidant composition of some natural extracts, causing nutritional losses. The drying step of the lyophilization process can potentially damage bioactive compounds by the disruption and/or elimination of the hydrogen bonding network of water molecules. These dehydration-induced stresses are also present during spray drying, and particularly high temperatures can be damaging to polyphenols. Another common problem is the sticking of the processed material to the walls of the drier, which deteriorates the yield of the products. To protect bioactive molecules from the stresses experienced during lyophilization and spray drying and for improved stability during subsequent storage in the dried state, biopharmaceuticals are formulated with a variety of excipients, support materials and coating agents. Thus, the obtained formulations, sometimes referred also as encapsulates, provide not only improved stability against negative effects of light, oxygen, moisture, but some other benefits, such as covering the bitter taste and astringency of polyphenols and improving the digestive stability [3,4].

This study aims to compare freeze drying with spray drying technology for the preservation of *T. serpyllum* extracts at laboratory scale. Since the main aim of encapsulation is the protection of active compounds from environmental conditions and, thus, the increase in the stability of products and the controlled delivery of encapsulated substances, the choice of packaging material represents an essential step [3]. In the present study, special attention is focused on the impact of gelatin as a wall material on process yield, the retention of polyphenols after drying and powder properties. This animal protein with GRAS (generally recognized as safe) status has been widely used as a good ingredient for powder formulation due to its biocompatibility, biodegradability, high water solubility and low cost. Gelatin increases the thermal stability of the product during freezing and primary drying of the lyophilization process [5,6]. Gelatin is also a good choice as a wall material in spray drying due to its good properties of emulsification, film-formation and an affinity to form a dense network [7,8].

Since the extraction efficiency and chemical profile of the extract are dependent on the extraction method applied, starting liquid extracts in this study are obtained by using maceration and heat-, ultrasound-, and microwave-assisted extraction technologies (ME, HAE, UAE, and MAE, respectively) under optimal conditions determined elsewhere [9]. The final goal of this study is to declare the optimal extraction method combined with the optimal drying technology for the production of *T. serpyllum* extract powder.

## 2. Results and Discussion

The lyophilized and spray-dried *T. serpyllum* extracts and gelatin-encapsulated extracts obtained by processing 100 g of the raw drug material were analyzed in terms of dried product yield, total polyphenol, flavonoid, sugar and peptide contents, retention efficiency (RE), chemical characteristics (Fourier-transform infrared spectroscopy, FTIR), antioxidant capacity (ABTS and DPPH assays), morphology (scanning electron microscopy, SEM), size distribution (laser diffraction spectroscopy), zeta potential (photon correlation spectroscopy), bulk density and solubility (pharmacopeia’s methods), and in vitro release of polyphenols.

### 2.1. Product Yield, Total Polyphenol, Flavonoid, Sugar and Peptide Contents and Retention Efficiency

The yield of dried extracts and gelatin encapsulates varied depending on the applied extraction and drying/encapsulation techniques, as shown by Table 1 and Table 2. Powder yield is given by two parameters. The first one, total powder yield (PY_T_), is expressed as the percentage ratio between the total mass of the obtained powder and the mass of the drug fed to the system (dry basis) prior to extraction. This one reflects total mass losses taking into account extraction and drying processes. Namely, the first mass loss occurred during the extraction process since extractable components represent only a portion of the weight of drug taken. Then, a certain mass loss happened after the drying process due to the sticking of the material to the walls of the drying chamber, but also some amount is lost during handling with the obtained light and fluffy powders. These mass losses assigned to the drying process are taken into account by powder yield after drying (PY_D_), which is calculated as the percentage ratio between the total mass of the obtained powder and the mass of the drug (dry basis) after extraction and prior to drying. According to our results, lower yields were obtained by spray drying than by freeze drying due to a severe deposition of the material on the cyclone walls of the spray dryer (Supplementary Figure S1). Total powder yield is higher for the samples obtained by MAE (18.9 and 17.7% for lyophilization and spray drying, respectively) compared to those produced by other extraction methods (12.9–14.1% and 8.7–11.7% for lyophilization and spray drying, respectively). Namely, according to the literature, microwaves lead to significant destruction of plant structure and consequent release of large amounts of ballast substances (proteins, sugars and lipids) into the extraction medium [10], which results in the higher dry weight content at the end of the extraction process, and higher total powder yield (expressed per gram of the drug prior to extraction) upon drying. For the sake of evidence, total sugar and total protein contents of the processed extracts are shown in Table 3. The PY_D_ of freeze-dried samples varied in the range 41–49%, while that of spray-dried extracts was in the range 30–34%. Generally, the yield of spray drying at laboratory scale with conventional spray dryers is 20–70% due to the loss of product in the walls of the drying chamber and the low capacity of the cyclone to separate fine particles (below 2 μm). The yield of freeze drying is usually very high, even above 90%. However, it should be stressed that this whole study on the evaluation of freeze vs. spray drying refers to the processing of a very small quantity of the raw drug material, only 100 g.

The resulting powders contained total polyphenols in the amount ~190 mg GAE per gram of powder, irrespective of the drying method applied, with somewhat higher values for the formulations obtained by HAE and lower values for those obtained by UAEp (ultrasound probe). According to the values of retention efficiency, dry formulations exhibited a loss of ~12.4 and ~34% (average values for all samples except MAE) of polyphenol compounds compared to the liquid counterparts, owing to freeze and spray drying, respectively. Namely, lyophilization and spray drying cause a reduction in polyphenolic glycosides relative to their content in fresh plant material [11]. The reduction in TPC in microwave extracts was significantly higher, 52.4% after lyophilization and 57.7% after spray drying, which can be attributed to higher sugar content in the extract and its adhesiveness (Table 2).

The TFC of the powder was dependent on both extraction and drying methods. Better recovery of flavonoids was achieved by lyophilization compared to spray drying, and worse by MAE compared to other extraction methods. This is not a surprising result bearing in mind the thermolability of flavonoids and high processing temperature of MAE (200 °C) and spray drying (inlet temperature of 140 °C).

In order to obtain a higher yield of extract and to increase the stability of bioactive extract components, 5% (*w*/*w*) of gelatin was added to the liquid extracts prior to exposing them to the drying process. According to the literature, this particular concentration allows the formation of aqueous solutions with reasonable viscosity in order to maintain proper atomization and efficient drying of the powder during the spray drying process [12]. PY_T_, PY_D_, TPC and TFC expressed per gram of powdered gelatin encapsulates are shown in Table 1. PY_T_ increased to ~32–35% and ~18–22% for lyophilized and spray-dried formulations, respectively, which meant that extract recovery (15–19% for lyophilization and 11–16% for spray drying), calculated by disregarding recovered gelatin (not shown), increased by a factor of ~1.3 due to the presence of gelatin. This increase can be attributed to the less sticky nature of the formulations containing gelatin and consequently their diminished deposition on the walls of the dryer chambers compared to extract alone (Supplementary Figure S1). The PY_T_ of pure gelatin determined by us was 47%, which is exactly the same value as the one reported by Wang in his doctoral thesis [12] for the same processing conditions as herein; this author also recommended an increase in inlet temperature up to 170 °C as a strategy for diminishing product loss manifested as wet deposits of semi-wet particles on the chamber wall and cyclone, but with a precaution that exposing the material to excessive temperatures might cause thermal degradation. The TPC of the obtained formulations with gelatin contained 62–75 and 53–74 mg GAE per gram of powder produced by lyophilization and spray drying, respectively, which corresponded to 290–360 and 204–228 mg GAE per gram of extract recovered by encapsulates produced by lyophilization and spray drying, respectively. When comparing to the TPC of the gelatin-free formulations, an increase of ~70% on average can be noticed in the case of lyophilization, solely due to the protective action of gelatin, and only of ~10% in the case of spray drying. The protective effect of gelatin was less manifested in terms of TFC per gram of extract recovered, since a rise of 27% on average was determined for lyophilized gelatin encapsulates compared to gelatin-free parallels, with no obvious effect on spray-dried formulations. Notable higher TPC was observed for the samples obtained by MAE compared to those obtained by other extraction methods, since MAE resulted in the highest extraction efficiency among all tested extraction methods; for the sake of comparison, the liquid MAE sample contained 45 mg GAE/L of TPC vs. 27 mg GAE/L in the ME sample as an example [9].

RE is defined as the amount of TPC recovered upon the drying/encapsulation process by lyophilization or spray drying, relative to the amount of TPC fed to the drying chamber. RE_TPC_ (retention efficiency of polyphenols) and RE_TFC_ (retention efficiency of flavonoids) were higher for lyophilized formulations compared to spray-dried analogues, which corresponds with the PY_T_ achieved by the methods (Table 1). Despite the fact that MAE dry samples exhibited the highest TPC and TFC, their RE_TPC_ and RE_TFC_ are actually the lowest among all. Although the liquid MAE formulation contained much more polyphenols compared to other liquid samples, obviously many of these polyphenolic compounds were lost upon drying; presumably, the more concentrated (in terms of TPC, TFC, proteins and sugars) liquid MAE sample had lower affinity toward gelatin. Furthermore, the presence of ballast and interfering substances in the microwave extract (caused by the degrading effects of microwaves) could be responsible for the lowest RE.

### 2.2. Antioxidant Capacity (ABTS and DPPH Assays)

According to the results of the ABTS assay, neither the extraction nor drying method expressed a significant difference between gelatin-free samples (Table 2), which is in accordance with TPC values (Table 1). Perhaps each of the used extraction technologies involves some issue unfavorable for polyphenols and its antioxidant capacity, but at the end all these ended with the same antioxidant capacity. HAE causes the degradation of polyphenol antioxidants, such as thermosensitive flavonoids and phenolic acids [3]. Ultrasound is known to boost polyphenol yield due to mechanical and thermal effects, but a local increase in temperature and the formation of free radicals by ultrasound waves lead to the degradation of a certain percentage of antioxidant compounds [13,14]. As regarding MAE, high internal pressure and temperature lead to an increase in the solubility of bioactive compounds in the extraction medium, but may reduce the antioxidant capacity of the extracts [15,16].

However, in the DPPH assay, the lyophilized extracts achieved better antioxidant activity (Table 2); it seems that the high temperature of spray drying caused a significant degradation of thermolabile antioxidant components responsible for hydrogen or electron donors to the free DPPH radical, such as flavonoids (Table 1), which is in accordance with the literature [17].

As regarding the ABTS radical scavenging activity of encapsulates, lyophilization resulted in insignificantly higher values compared to spray drying, while MAE resulted in a higher value compared to other samples, which correlates with TPC and TFC (Table 1 and Table 2). On the other hand, in the DPPH assay there is not any systematic dependence of antioxidant potential on the extraction or drying method.

### 2.3. Chemical Characteristics (FTIR)

**Figure 1:**
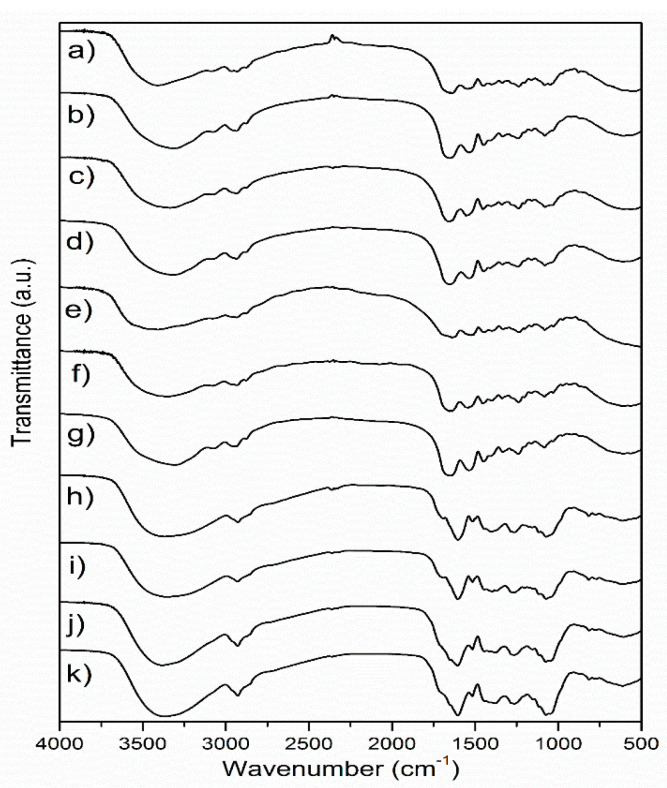
FTIR spectra of (**a**) freeze-dried HAE encapsulate, (**b**) spray-dried HAE encapsulate, (**c**) freeze-dried MAE encapsulate, (**d**) spray-dried MAE encapsulate, (**e**) raw gelatin, (**f**) freeze-dried gelatin, (**g**) spray-dried gelatin, (**h**) freeze-dried HAE extract, (**i**) spray-dried HAE extract, (**j**) freeze-dried MAE extract, (**k**) spray-dried MAE extract; HAE and MAE, heat- and microwave-assisted extraction, respectively.

FTIR analysis of the samples was performed in order to examine the influence of different extraction and drying methods on the chemical characteristics of wild thyme extracts and gelatin encapsulates. The FTIR spectra of freeze and spray-dried HAE and MAE extracts and the corresponding encapsulates, as well as of raw and dried gelatin, are shown in Figure 1.

In the FTIR spectra of lyophilized and spray-dried extracts obtained by different extraction methods (Figure 1), several bands with pronounced intensity can be observed, such as a band at about 1604 cm^−1^, which probably originates from COO- vibrations in polysaccharide molecules [18], while the band at about 1072 cm^−1^ is close to the positions of the band originating from C-OH vibrations present in the carbohydrates [19]. The band at about 2927–2929 cm^−1^ originates from C-H vibrations and corresponds to the same vibration identified in green tea extract [20]. The largest variation between spectra of the extracts was observed in the positions of the bands originating from the vibrations of the OH groups (spectral region 3000–3600 cm^−1^). These differences can primarily be related to the drying conditions, but also to the chemical composition of the extracts, which also depends on the extraction method. The band found at 1516 cm^−1^ in all extracts most likely originates from the extracted flavonoids [21]. The band at 1265 cm^−1^ can be related to the polyphenolic compounds, C-C-O vibrations; a band of lower intensity at about 817 cm^−1^ could be related to the present monoterpenes, C-H vibrations [18]. The variations in the FTIR spectra of the extracts obtained by different extraction procedures are relatively minor. A band with a maximum at about 1400 cm^−1^ is moved to 1377 cm^−1^ in the HAE sample, and it has been associated with the presence of monoterpenes [18]. The band at about 1400–1404 cm^−1^ could originate from carboxylates [22].

Several characteristic bands were observed in the FTIR spectra of gelatin, and the most important bands were related to the protein structure: 1637 cm^−1^, amide I-C=O vibrations and 1541 cm^−1^, amide II-N-H vibrations [23,24]. The band with a maximum at about 2935 cm^−1^ originates from C-H tensile vibrations, while bands at 1448 cm^−1^, 1332 cm^−1^ and 1080 cm^−1^, which are described in the literature for gelatin, most likely originate from C-O vibrations [24] or from carboxyl groups from amino acids [25]. The amide III band (N-H vibrations) can be observed at about 1234 cm^−1^ [23]. Changes in the FTIR spectra of the carrier in comparison to the starting (native) gelatin are noticeable primarily in the area of vibrations of -OH groups (3000–3700 cm^−1^), which is probably a consequence of structure disturbance and intermolecular bonds occurring in the drying process. Other changes are in the spectral region of the amide bands, where changes in the position of the amide I band (new position at about 1654 cm^−1^) are observed, as well as changes in the position of the amide II band (new position at 1558 cm^−1^) in freeze-dried gelatin. Schwegman et al. [26] showed that lyophilization and freezing can affect the secondary structure of proteins, which was observed through changes in the position of the amide I band. The addition of glucose, maltose, mannitol, sucrose or dextran can reduce the impact of lyophilization on the protein structure [26,27]. Regarding the differences between the influence of freeze and spray drying on the structure of gelatin, changes are visible in the positions of the bands originating from OH groups, as well as in the position of the amide II band, which is in accordance with the literature [28]. After encapsulation, the FTIR spectra of the samples obtained by lyophilization and spray drying almost exclusively showed bands originating from the carrier, i.e., gelatin (Figure 1), which is, according to the literature, indicative for efficient wrapping of active components [29].

### 2.4. Morphological Characteristics, Particle Size Distribution and Zeta Potential

**Figure 2:**
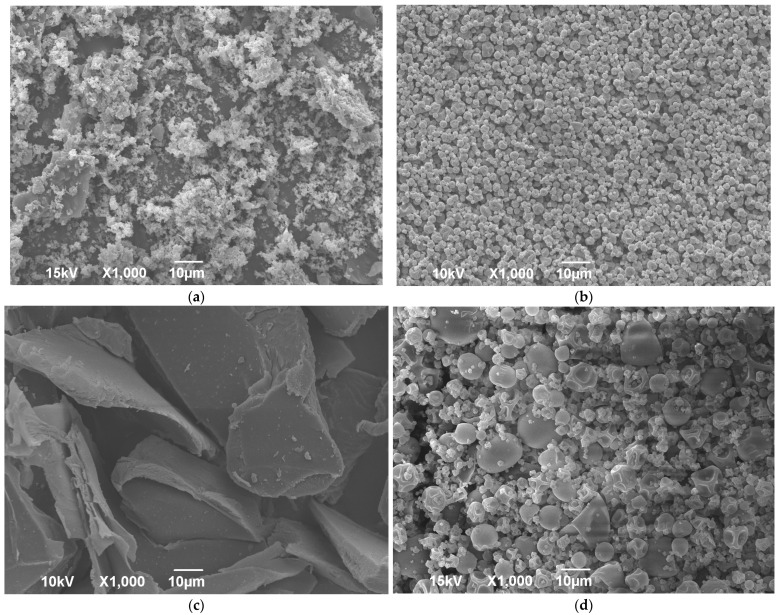
SEM images of freeze-dried (**a**,**c**) and spray-dried (**b**,**d**) wild thyme extracts (**a**,**b**) and encapsulated extracts (**c**,**d**) obtained in heat-assisted extraction.

All dry extract formulations appeared as yellowish powders compared to white powders obtained by processing pure gelatin (Supplementary Figure S2). The SEM photos of lyophilized and spray-dried pure wild thyme extracts and gelatin encapsulates (HAE sample as representative) are shown in Figure 2. A comparison of the images revealed a notable variation with regard to particle structure and size allotment amongst different products, as also confirmed by laser light diffraction analysis (Table 4). Freeze drying of the extract resulted in uneven and brittle particles crushed in a voluminous powder (Figure 2a). Freeze-dried encapsulate presented an irregular shape like broken glass with a smooth surface (Figure 2c) and large particles, with size d_50_ ranging between 130 and 223 µm depending on the sample (Table 4). On the other hand, spray drying formed small spherical and pseudo-spherical particles with an uneven surface, much smaller and more uniform in the case of blank extract (Figure 2b) than formulations with gelatin (Figure 2d). The concavity and wrinkles of spray-dried particles have been associated with the rapid evaporation of moisture during the process [30]. A possibility for the production of micro-spheres with a smoother outer surface should be looked for in the addition of sucrose or mannitol [31,32]. The average diameter d_50_ of the spray-dried formulations containing gelatin was about 8 µm, irrespective of the type of sample, while size distribution was quite wide, characterized by high SPAN values (~2) (Table 4).

The zeta potential of all formulations (extracts and encapsulates) was determined to reflect the physicochemical stability (Table 4). All samples exhibited negative surface charge, but with low zeta potential values showing colloidal instability due to insufficiently high repulsive forces. The values are more negative for blank extract particles than for encapsulates, and the last are rather close to the surface charge of pure gelatin (−3.4 ± 0.2 mV and −1.9 ± 0.3 mV for lyophilized and spray-dried particles, respectively), which protonates (via carboxylic group of lysine and arginine residues) at a pH below their isoelectric point (pH 6.1), influencing overall zeta potential. According to the literature, the zeta potential of 5% aqueous gelatin solution goes from positive (+21 mV) to negative (−12 mV) with increasing pH from 2 to 10 [12]. Therefore, a possible solution for the problem of the instability of dry gelatin particles can be found in adjusting the pH of the gelatin solution to 3 prior to drying to achieve maximum surface charge. Notable lower surface charge was detected for MAE samples compared to the parallels produced by other extraction methods.

### 2.5. Bulk Density and Solubility

The pharmacopoeia procedures were used to determine the bulk density and solubility of dry wild thyme extract formulations. These properties are important from the aspect of their application, for the transport, storage, packaging, and mixing processes and for reconstitution. The bulk densities of the lyophilized and spray-dried powders ranged between 0.09 and 0.2 g/cm^3^ and between 0.16 and 0.28 g/cm^3^, respectively, with the significantly higher values of the spray-dried samples compared to their lyophilized parallels (Table 4). The reason lies in better and more compact packaging of morphologically more regular and smaller spray-dried particles, as evidenced by SEM (Figure 2) and laser particle size analysis (Table 4). Pure extract powders mainly expressed higher values than the encapsulate counterparts since the carrier per se had low bulk density (lyophilized 36.0 ± 0.2 g/mL and spray-dried 150.0 ± 0.3 g/mL). Solubility is also considerably better for the pure extract powders as compared to the encapsulates, and for the freeze-dried vs. spray-dried encapsulates; the last issue can be associated with the tendency for aggregation characteristic for the small particles of regular shape, such as spray-dried microspheres in contrast to large particles of irregular shapes typical for lyophilized powders, which do not express this affinity so much. According to the literature, aggregation may affect the particle dissolution rate [33]. Once again, the MAE pure extract was distinguished from the others by a higher bulk density and lower solubility due to the aforementioned higher amount of sugars and proteins (Table 3).

### 2.6. Thermal Characteristics

In order to determine the thermal stability of wild thyme extracts and their gelatin encapsulates, as well as the interactions between encapsulated components and gelatin, the DSC technique was employed. Besides, the DSC method is useful to monitor changes in the thermal characteristics of the carrier after the encapsulation of the target compounds. DSC thermograms of lyophilized and spray-dried gelatin extracts obtained by HAE and MAE and their gelatin encapsulates are shown in Supplementary Figure S3. The values of the peak transition temperature and the enthalpy change (∆H) are shown in Table 5.

As can be seen from DSC thermograms (Supplementary Figure S3), all samples show a large and broad endothermic peak in the 50–150 °C range, which is probably the sum of glass transition and melting [34]. The peak maximum of this thermal event is positioned at higher temperatures in the case of the spray-dried samples compared to the freeze-dried counterparts, which is also accompanied with the higher enthalpy change, indicating higher thermal stability. The transition temperature of the processed pure gelatin (80 and 89.5 °C for freeze and spray-dried gelatin, respectively) appeared consistent with the literature [35]. Generally, glass transition temperature is affected by several factors, such as the chemical structure, molecular weight, and moisture content of the material [36]. The higher transition temperature of spray-dried samples indicates higher moisture content compared with that of the freeze-dried parallels. As a consequence of adding carrier material, the final transition temperatures of encapsulates increased from the values of pure extracts, and basically Tg is related to the molecular weight of the polymer present in this material (the Tg increases with increasing molecular weight), as well as water content. Furthermore, the MAE extract exhibited higher stability (higher value of peak maximum and enthalpy change) compared to the HAE parallel, which can be associated with the specific chemical composition (Table 3). Namely, carbohydrates (present in the MAE samples in a higher amount than in HAE) preserve proteins’ secondary structure, as reported by other authors [26,27].

### 2.7. Polyphenol Release Kinetics

**Figure 3:**
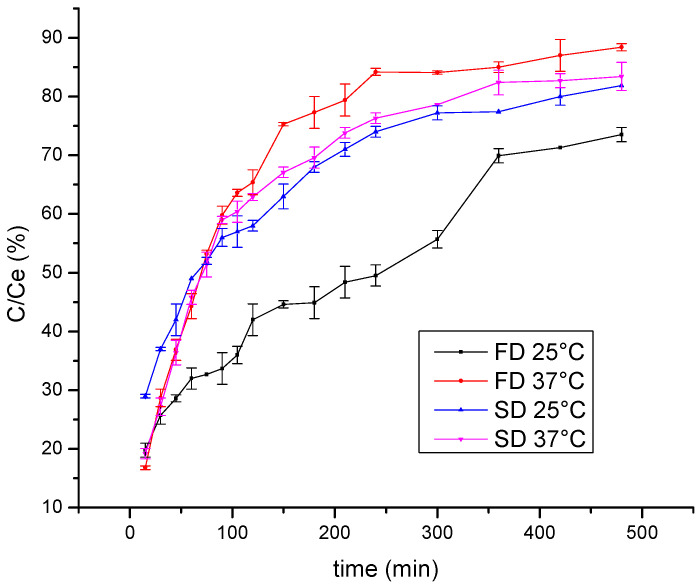
Kinetics of polyphenol release from freeze-dried (FD) and spray-dried (SD) HAE wild thyme extract encapsulates, monitored at 25 °C and 37 °C; C, polyphenol concentration at the time of measurement; Ce, the equilibrium polyphenol concentration; HAE, heat-assisted extraction.

One of the aims of polyphenol encapsulation is to obtain products with a prolonged release of active compounds. Thus, the release of wild thyme polyphenols from lyophilized and spray-dried gelatin encapsulates was examined using the Franz diffusion cell. The kinetics of polyphenol release monitored at room temperature and at 37 °C are shown in Figure 3. The results are presented as the dependence of C/Ce on time, where C is the polyphenol concentration at the time of measurement and Ce is the equilibrium polyphenol concentration. Expectedly, a higher release rate was observed at 37 °C compared to 25 °C so that after 1 h about 45% was released at room temperature and about 60% at 37 °C. Furthermore, a statistically significant difference was observed between freeze and spray-dried encapsulates at 25 °C, such that the spray-dried encapsulates released polyphenols more rapidly with the diffusion coefficient 3.61∙10^−9^ m^2^/s vs. 2.34∙10^−9^ m^2^/s obtained for the lyophilized sample. This result can be explained by a higher specific surface area of small microspheres in spray-dried powder compared to that of irregular shapes characteristic for freeze-dried powder. By comparing curves in Figure 3 with the diffusion rate of polyphenols from gelatin-free formulations (data not shown), a retarding effect was observed due to a coating material, in such a manner that steady state was reached after approximately 360 min vs. only 75 min needed for free polyphenols to reach a concentration plateau.

## 3. Materials and Methods

### 3.1. Plant Material and Reagents

Plant material used for preparation of polyphenol extracts was *T. serpyllum* herb from the Institute for Medicinal Plants Research “Dr Josif Pančić”, Serbia. Used reagents were ethanol and sodium carbonate (Fisher Scientific, Loughborough, UK), potassium persulfate (Centrohem, Stara Pazova, Serbia), Folin–Ciocalteu reagent and gallic acid (Merck, Darmstadt, Germany), sodium nitrite (Alkaloid, Skopje, Macedonia), sodium hydroxide (Alfapanon, Bački Petrovac, Serbia), aluminum chloride, catechin, 2,2′-azino-bis(3-ethylbenzothiazoline-6-sulphonic acid) or ABTS, 6-hydroxy-2,5,7,8-tetramethylchroman-2-carboxylic acid or Trolox, and 2,2-diphenyl-1-picrylhydrazyl or DPPH (Sigma-Aldrich, St. Louis, MO, USA). Beefskin gelatin—220 bloom (Gelnex, Santa Catarina, Brazil) was used as a carrier.

### 3.2. Preparation of Extracts

Polyphenol extracts from *T. serpyllum* were prepared using five extraction techniques: maceration (ME), heat-assisted extraction (HAE), extraction by using ultrasound probe (UAEp), extraction in ultrasound bath (UAEb), and microwave-assisted extraction (MAE). ME was performed on the shaker Unimax 1010 (Heidolph, Schwabach, Germany), using plant particle size of 0.3 mm, 50% ethanol as an extraction medium and 1:30 solid to solvent ratio, during 15 min, at room temperature. Samples in HAE were obtained at 80 °C in the incubator shaker KS 4000i control (IKA, Königswinter, Germany), using the same extraction parameters as in the case of ME. Further, ultrasound probe applied in the next extraction procedure was the ultrasonic processor of 750 W output with 20 kHz converter (Sonics, Newtown, PA, USA) and a solid titanium probe of 19 mm diameter at 80% of amplitude (the same factor levels as in previous cases). The extraction in ultrasound bath Sonorex Digitec (Bandelin electronic, Berlin, Germany) was performed using particle size of 0.3 mm, 30% ethanol and 1:30 solid to solvent ratio, during 30 min. MAE was carried out at 200 °C using particles of 0.3 mm, 48% ethanol and 1:25 solid to solvent ratio, during 86 s, in microwave reactor Monowave 300 (850 W, Anton Paar, Graz, Austria). The extracts were made in triplicate for each extraction procedure.

### 3.3. Preparation of Freeze and Spray-Dried Gelatin-Encapsulated Extracts

In order to evaporate ethanol from liquid extracts, the samples were placed in rotary evaporator Heizbad Hei-VAP (Heidolph, Berlin, Germany) at 40–50 °C, pressure of 50 mbar and rotation speed of 150 rpm. Subsequently, 50 mL of liquid extract without ethanol was mixed with 2.5 g of gelatin and the mixture was stirred on the magnetic stir plate, RTC basic (IKA, Königswinter, Germany), at 40 °C for approximately 15 min.

Before the lyophilization, the samples (dissolved gelatin, pure extracts and extracts with 5% *w*/*w* of gelatin) were frozen in freezer, LAB11/EL19LT (Elcold, Hobro, Denmark), at −80 °C for 1 h. In freeze dryer Beta 2–8 LD plus (Christ, Osterode am Harz, Germany), main drying was carried out at −75 °C and pressure of 0.011 mbar for 24 h and final drying at −65 °C and pressure of 0.054 mbar for 1 h.

Spray drying of dissolved gelatin, pure extracts and extracts with 5% *w*/*w* of gelatin was performed in Mini Spray Dryer B-290 (Büchi, Flawil, Switzerland) equipped with nozzle of 0.7 mm. The inlet temperature was 140 °C, while outlet temperature was 72–75 °C. The air flow rate was 45 mm and the rate of liquid feed was 30%.

### 3.4. Determination of Total Polyphenol, Flavonoid, Sugar and Peptide Contents

TPC in pure and gelatin-encapsulated extracts was determined using Folin–Ciocalteu method given by Li et al. [37]. The results are expressed as milligrams of GAE per gram of dried or encapsulated extract (mg GAE/g). TFC in pure and gelatin-encapsulated extracts was determined by a colorimetric assay as described by Petrović et al. [38]. The results are expressed as milligrams of catechin equivalents per gram of dried or encapsulated extract (mg CE/g). In order to determine total sugar content in pure lyophilized and spray-dried extracts, a simple colorimetric assay with phenol–sulfuric acid was used [39]. Bradford method, as rapid and accurate assay, was used for the estimation of peptide concentration in pure lyophilized and spray-dried extracts [40]. The results of total sugar and peptide content were expressed as percentage. All absorbance readings were performed in UV spectrophotometer, UV-1800 (Shimadzu, Kyoto, Japan).

### 3.5. Powder Yield and Retention Efficiency (RE)

Powder yield is calculated by two equations.

The total powder yield, PY_T_, is the product recovery given by the percent ratio between the total mass of product recovered, m_p_, by the mass of drug prior to extraction (dry basis), m_dw_:

(1) $${\mathrm{PY}}_{T}=\frac{m_{p}}{m_{\mathrm{dw}}}$$

The powder yield after drying, PY_D_, is given by the percent ratio between the total mass of product recovered, m_p_, by the non-solvent mass of the infeed solution, m_is_:

(2) $${\mathrm{PY}}_{D}=\frac{m_{p}}{m_{\mathrm{is}}}$$

Determination of RE was performed after dissolution of dried extracts and gelatin-encapsulated extracts and sonication during 5 min. The dispersion was filtered through a membrane filter and TPC or TFC in the clear solution was determined spectrophotometrically (described in previous paragraph). The retention efficiency of TPC was calculated using the following formula:

(3) $${\mathrm{RE}}_{\mathrm{TPC}}\left[\%\right]=\frac{{\mathrm{TPC}}_{p}}{{\mathrm{TPC}}_{\mathrm{is}}}\times 100$$

where TPC_p_ represented TPC in powder and TPC_is_ in infeed solution.

The retention efficiency of TFC was calculated using the following formula:

(4) $${\mathrm{RE}}_{\mathrm{TFC}}\left[\%\right]=\frac{{\mathrm{TFC}}_{p}}{{\mathrm{TFC}}_{\mathrm{is}}}\times 100$$

where TFC_p_ represented TFC in powder and TFC_is_ in infeed solution.

### 3.6. Fourier-Transform Infrared Spectroscopy (FTIR)

FTIR analysis of gelatin as a carrier and pure and encapsulated extracts was performed using FTIR spectrophotometer, IRAffinity-1 (Shimadzu, Kyoto, Japan). The samples were prepared as KBr pellets and analyzed in the spectral range 4000–500 cm^−1^, with a resolution of 4 cm^−1^.

### 3.7. Antioxidant Activity (ABTS and DPPH Assays)

The antioxidant activity of freeze and spray-dried pure and gelatin-encapsulated extracts was analyzed according to ABTS and DPPH methods previously published by Re et al. [41] and Horžić et al. [42], respectively. In ABTS assay, the antioxidant activity was expressed as mmol of Trolox equivalents per gram of pure or encapsulated extract (mmol Trolox/g), while in DPPH test the results were expressed as IC_50_—the concentration required to scavenge 50% of free DPPH radicals (mg/mL).

### 3.8. Scanning Electron Microscopy (SEM), Particle Size Distribution and Zeta Potential

Microstructure analysis of freeze and spray-dried gelatin and pure and encapsulated extracts was carried out using SEM, JSM-6390LV (JEOL, Tokyo, Japan). Before the SEM analysis, the samples were coated with gold on sputter coater, SCD 005 (BAL-TEC, Balzers Liechtenstein, Germany). The size of dried particles and size distribution were determined using laser particle size analyzer, Mastersizer 2000 (Malvern Instruments, Malvern, UK). The width or SPAN factor of the size distributions was calculated using the equation: (d_90_-d_10_)/d_50_, d_10_—10% of the particles smaller than this diameter, d_50_—50% of the particles smaller than this diameter, and d_90_—90% of the particles smaller than this diameter. Zeta potential of dried particles was measured using photon correlation spectroscopy, in Zetasizer Nano Series and Zetasizer Software (Malvern, Malvern, UK).

### 3.9. Determination of Bulk Density and Solubility

Bulk density and solubility of the lyophilized and spray-dried extracts and gelatin-encapsulated extracts were examined following the method described in The European Pharmacopeia [43]. Bulk density was determined by placing 30 g of powder into a graduated glass cylinder of 100 mL and expressed as g/mL. In order to investigate solubility of samples, 0.5 g of dried sample was dissolved in 50 mL of water and continuously stirred on the magnetic stir plate at 150 rpm during 30 min, at room temperature. Subsequently, the samples were centrifuged at 3000 rpm during 5 min and the supernatants were dried at 105 °C until constant weight. Based on the obtained and initial mass of the samples, the solubility of the extracts and gelatin encapsulates was calculated and the results were expressed as percentage.

### 3.10. Differential Scanning Calorimetry (DSC)

Thermal properties of dried gelatin, selected lyophilized and spray-dried pure extracts (obtained in HAE and MAE) and their encapsulates were analyzed by DSC131 Evo (SETARAM Instrumentation, Caluire-et-Cuire, France). The samples were placed in aluminum pans (30 μL), which were subsequently hermetically sealed. An empty pan was used as a reference. The heating profile was set as follows: both pans (reference and sample) were stabilized at 30 °C for 5 min then heated to 300 °C, with a heating rate of 10 °C/min, while the nitrogen flow was 20 mL/min. A baseline run was performed using empty pans under the same conditions, whereas baseline subtraction and determination of enthalpy (J/g) were carried out by CALISTO PROCESSING software equipped by SETARAM Instrumentation.

### 3.11. In Vitro Polyphenol Release Study

Polyphenol release from selected freeze and spray-dried pure extracts (obtained using HAE) and the encapsulates thereof was performed in Franz diffusion cell (PermeGear, Pennsylvania, USA), at 25 °C and at 37 °C. The sample was placed in the donor section, whereas the receptor section was filled with 20 mL of water and constantly mixed. Two mentioned sections were separated by acetate–cellulose membrane. The samples were taken in appropriate time intervals during 4 h for pure extracts and during 24 h for encapsulated extracts. Polyphenol concentration was determined spectrophotometrically using previously mentioned Folin–Ciocalteu method.

The results of the release study were used for further determination of polyphenol diffusion coefficients and diffusion resistances of pure and encapsulated extracts. The diffusion coefficients were determined using Fick’s second law:

(5) $$D\beta t=\ln\left(\frac{c_{d}^{0}-c_{r}^{0}}{c_{d}-c_{r}}\right)$$

where D was the diffusion coefficient, β was a geometrical constant, c_d_ and c_r_ were concentrations of polyphenols in donor and receptor sections, respectively, at time *t*, whereas c_d_^0^ and c_r_^0^ were polyphenol concentrations at *t* = 0. Additionally, diffusion resistance (R) was calculated according to the equation: R = δ/D, where δ is membrane thicknesses.

### 3.12. Statistical Analysis

In the present study, statistical analysis was carried out by STATISTICA 7.0; the statistical significance was determined using analysis of variance, followed by Duncan’s post hoc test (the differences were considered statistically significant at *p* < 0.05).

## 4. Conclusions

In this study, different extraction methods were used to obtain liquid wild thyme extracts which were then, with or without gelatin, subjected to either freeze or spray drying for their preservation. MAE gave a formulation with a higher quantity of ballast materials, which caused a higher loss of TPC as a consequence of the drying process, but ended with higher PY_T_ after drying. Both drying techniques and all tested extraction methods resulted in satisfying dry extract gelatin-based formulations considering release and physical properties. Gelatin as a coating material increased extract recovery by diminishing the deposition of the material on the walls of the drying chamber and provided TPC protection, particularly significant in the case of freeze drying. At laboratory scale, freeze drying when compared with spray drying resulted in a higher total powder yield and lower TPC and TFC loss due to drying, which corresponded with the antioxidant activity of the formulations. Despite the more appealing morphology and higher bulk density of spray-dried powders, freeze-dried formulations provided a longer release of polyphenols and better solubility.

## Figures and Tables

**Table 1:** Total powder yield (PY_T_), powder yield after drying (PY_D_), total polyphenol content (TPC) and total flavonoid content (TFC), retention efficiency of polyphenols (RE_TPC_) and retention efficiency of flavonoids (RE_TFC_) of wild thyme extracts and gelatin encapsulates produced by freeze drying (FD) and spray drying (SD).

	PY_T_ (%)		PY_D_ (%)		TPC (mg GAE/g)		TFC (mg CE/g)		RE_TPC_ (%)		RE_TFC_ (%)	
	FD	SD	FD	SD	FD	SD	FD	SD	FD	SD	FD	SD
Extract Sample												
ME	12.9 ± 0.6 ^b,1^	9.9 ± 0.9 ^bc,2^	41.1 ± 2.3 ^b,1^	31.4 ± 2.7 ^a,2^	187.7 ± 1.7 ^b,1^	190.8 ± 3.9 ^b,1^	110.4 ± 0.5 ^b,1^	100.3 ± 1.3 ^b,2^	88.8 ± 2.2 ^b,1^	70.3 ± 1.4 ^a,2^	90.1 ± 3.1 ^a,1^	61.9 ± 2.2 ^a,2^
HAE	13.8 ± 0.3 ^b,1^	11.7 ± 1.5 ^c,2^	40.4 ± 3.4 ^b,1^	33.9 ± 1.6 ^a,2^	195.5 ± 1.4 ^a,1^	200.4 ± 4.4 ^a,1^	113.7 ± 0.6 ^a,1^	104.0 ± 0.5 ^a,2^	86.2 ± 2.3 ^b,1^	63.4 ± 2.6 ^b,2^	89.0 ± 1.5 ^a,1^	60.2 ± 1.3 ^ab,2^
UAEb	14.1 ± 1.5 ^b,1^	8.7 ± 1.2 ^b,2^	49.1 ± 4.2 ^a,1^	30.5 ± 0.5 ^a,2^	181.9 ± 1.5 ^c,1^	183.1 ± 1.2 ^c,1^	112.0 ± 1.0 ^a,1^	96.9 ± 0.5 ^c,2^	92.8 ± 3.8 ^a,1^	66.0 ± 2.2 ^b,2^	90.8 ± 2.4 ^a,1^	64.2 ± 1.0 ^a,2^
UAEp	13.8 ± 0.9 ^b,1^	9.3 ± 0.9 ^bc,2^	48.4 ± 1.2 ^a,1^	32.6 ± 2.3 ^a,2^	190.3 ± 1.3 ^b,1^	190.8 ± 1.6 ^b,1^	113.4 ± 1.1 ^a,1^	100.0 ± 1.5 ^b,2^	82.6 ± 4.2 ^bc,1^	64.3 ± 1.7 ^b,2^	85.1 ± 0.8 ^b,1^	61.7 ± 2.1 ^a,2^
MAE	18.9 ± 0.5 ^a,1^	17.7 ± 0.2 ^a,2^	48.8 ± 3.7 ^a,1^	34.3 ± 3.9 ^a,2^	190.2 ± 1.9 ^b,1^	190.9 ± 4.0 ^b,1^	98.4 ± 0.3 ^c,1^	90.9 ± 2.2 ^d,2^	47.6 ± 1.5 ^d,1^	42.3 ± 3.1 ^c,2^	45.1 ± 2.1 ^c,1^	44.3 ± 1.8 ^c,1^
Encapsulate Sample												
ME	35.9 ± 1.2 ^a,1^	20.6 ± 0.1 ^b,2^	53.4 ± 3.5 ^b,1^	40.8 ± 7.1 ^a,2^	65.0 ± 1.0 ^c,1^	53.3 ± 1.1 ^b,2^	27.8 ± 0.5 ^b,1^	23.7 ± 0.0 ^c,2^	51.6 ± 1.1 ^c,1^	48.7 ± 0.7 ^a,2^	54.1 ± 1.7 ^c,1^	43.9 ± 1.6 ^a,2^
HAE	34.9 ± 0.2 ^a,1^	21.7 ± 1.1 ^ab,2^	51.3 ± 7.2 ^b,1^	44.0 ± 4.5 ^a,1^	69.8 ± 2.0 ^b,1^	55.5 ± 0.3 ^b,2^	30.4 ± 1.6 ^a,1^	26.1 ± 0.6 ^b,2^	55.0 ± 0.9 ^b,1^	35.2 ± 1.5 ^c,2^	57.8 ± 0.2 ^b,1^	35.7 ± 1.2 ^c,2^
UAEb	35.2 ± 0.1 ^a,1^	21.1 ± 0.4 ^ab,2^	68.7 ± 6.3 ^a,1^	40.0 ± 4.7 ^a,2^	62.2 ± 0.4 ^d,1^	53.1 ± 1.3 ^b,2^	29.0 ± 0.9 ^ab,1^	23.2 ± 1.5 ^c,2^	62.7 ± 1.2 ^a,1^	43.5 ± 1.7 ^b,2^	61.4 ± 0.9 ^a,1^	41.9 ± 0.5 ^b,2^
UAEp	35.7 ± 0.6 ^a,1^	17.9 ± 1.2 ^c,2^	69.1 ± 3.6 ^a,1^	42.3 ± 5.8 ^a,2^	61.4 ± 1.2 ^d,1^	53.9 ± 1.3 ^b,2^	26.0 ± 0.9 ^c,1^	24.1 ± 0.8 ^c,2^	41.2 ± 0.5 ^d,1^	38.1 ± 0.9 ^c,2^	45.7 ± 1.0 ^d,1^	36.1 ± 0.6 ^c,2^
MAE	32.3 ± 0.6 ^b,1^	21.5 ± 0.1 ^a,2^	64.5 ± 2.7 ^a,1^	44.5 ± 5.5 ^a,2^	75.4 ± 0.8 ^a,1^	73.7 ± 0.0 ^a,2^	30.0 ± 1.3 ^a,1^	28.7 ± 0.5 ^a,2^	28.0 ± 1.4 ^e,1^	23.7 ± 1.7 ^d,2^	25.4 ± 1.7 ^e,1^	24.0 ± 1.3 ^d,1^

Values with the same letter (^a–e^) in each column and number (^1–2^) in each row showed no statistically significant difference (*p* > 0.05; n = 3; analysis of variance, Duncan’s post hoc test); ME, maceration extraction; HAE, heat-assisted extraction; UAEb, extraction in ultrasound bath; UAEp, extraction by ultrasound probe; MAE, microwave-assisted extraction; GAE, gallic acid equivalents; CE, catechin equivalent; IC_50_, n.a., not applicable.

**Table 2:** Antioxidant capacity of wild thyme extracts and gelatin encapsulates produced by freeze drying (FD) and spray drying (SD).

	ABTS (mmol Trolox/g)		DPPH, IC_50_ (mg/mL)	
	FD	SD	FD	SD
	Extract Sample			
ME	14.8 ± 1.3 ^a,1^	13.6 ± 1.6 ^a,1^	0.18 ± 0.00 ^b,1^	0.23 ± 0.00 ^b,2^
HAE	14.0 ± 0.7 ^a,1^	13.9 ± 1.0 ^a,1^	0.17 ± 0.00 ^ab,1^	0.22 ± 0.00 ^ab,2^
UAEb	14.3 ± 1.1 ^a,1^	13.3 ± 0.7 ^a,1^	0.17 ± 0.00 ^ab,1^	0.22 ± 0.00 ^ab,2^
UAEp	14.5 ± 1.1 ^a,1^	13.4 ± 0.9 ^a,1^	0.16 ± 0.02 ^a,1^	0.22 ± 0.00 ^ab,2^
MAE	13.9 ± 1.6 ^a,1^	14.1 ± 1.2 ^a,1^	0.17 ± 0.01 ^ab,1^	0.21 ± 0.02 ^a,2^
	Encapsulate Sample			
ME	4.2 ± 0.3 ^ab,1^	2.7 ± 0.9 ^b,2^	1.05 ± 0.12 ^b,1^	0.92 ± 0.02 ^ab,1^
HAE	4.3 ± 0.4 ^ab,1^	3.3 ± 0.7 ^b,1^	0.79 ± 0.06 ^a,1^	0.80 ± 0.04 ^a,1^
UAEb	3.6 ± 0.5 ^b,1^	3.0 ± 0.2 ^b,1^	0.98 ± 0.09 ^b,1^	1.04 ± 0.22 ^b,1^
UAEp	3.5 ± 0.2 ^b,1^	3.1 ± 0.3 ^b,1^	0.82 ± 0.02 ^a,1^	0.79 ± 0.02 ^a,1^
MAE	4.8 ± 0.8 ^a,1^	5.2 ± 0.3 ^a,1^	1.00 ± 0.05 ^b,1^	0.84 ± 0.18 ^a,1^

Values with the same letter (^a–b^) in each column and number (^1–2^) in each row showed no statistically significant difference (*p* > 0.05; n = 3; analysis of variance, Duncan’s post hoc test); ME, maceration extraction; HAE, heat-assisted extraction; UAEb, extraction in ultrasound bath; UAEp, extraction by ultrasound probe; MAE, microwave-assisted extraction; IC_50_, the concentration required to scavenge 50% of free DPPH radicals.

**Table 3:** Total sugar and peptide contents of wild thyme extracts produced by freeze drying (FD) and spray drying (SD).

	Total Sugars (%)		Total Peptides (%)	
Sample	FD	SD	FD	SD
ME	30.5 ± 0.0 ^b,2^	31.4 ± 0.3 ^b,1^	6.6 ± 0.2 ^bc,1^	6.7 ± 0.2 ^b,1^
HAE	27.3 ± 0.2 ^c,2^	31.4 ± 0.8 ^b,1^	6.9 ± 0.1 ^b,1^	6.3 ± 0.1 ^bc,2^
UAEb	30.0 ± 0.0 ^b,2^	31.4 ± 0.0 ^b,1^	5.2 ± 0.1 ^d,2^	5.7 ± 0.3 ^c,1^
UAEp	30.0 ± 0.6 ^b,2^	31.3 ± 0.5 ^b,1^	6.4 ± 0.1 ^c,1^	6.4 ± 0.2 ^b,1^
MAE	32.5 ± 1.0 ^a,2^	34.7 ± 0.4 ^a,1^	8.5 ± 0.2 ^a,1^	7.7 ± 0.3 ^a,2^

Values with the same letter (^a–d^) in each column and number (^1–2^) in each row showed no statistically significant difference (*p* > 0.05; n = 3; analysis of variance, Duncan’s post hoc test); ME, maceration; HAE, heat-assisted extraction; UAEb, extraction in ultrasound bath; UAEp, extraction by ultrasound probe; MAE, microwave-assisted extraction.

**Table 4:** Particle size d_50_, SPAN factor, zeta potential (ζ), bulk density and solubility of freeze-dried (FD) and spray-dried (SD) formulations of wild thyme extracts and gelatin encapsulates.

	d_50_ [µm]		SPAN		ζ (mV)		Bulk Density (g/mL)		Solubility (%)	
	FD	SD	FD	SD	FD	SD	FD	SD	FD	SD
Extract samples										
ME	N.D.	N.D.	N.D.	N.D.	−18.7 ± 0.8 ^a,1^	−18.3 ± 0.4 ^a,1^	125.0 ± 1.8 ^d,2^	200.0 ± 3.3 ^d,1^	80 ± 4 ^a,1^	84 ± 2 ^a,1^
HAE	N.D.	N.D.	N.D.	N.D.	−16.3 ± 0.1 ^b,1^	−14.7 ± 0.1 ^c,2^	140.0 ± 2.4 ^b,2^	235.5 ± 4.7 ^b,1^	78 ± 3 ^a,1^	76 ± 2 ^c,1^
UAEB	N.D.	N.D.	N.D.	N.D.	−17.1 ± 0.6 ^b,1^	−14.8 ± 0.0 ^c,2^	130.5 ± 1.0 ^c,2^	215.0 ± 4.1 ^c,1^	78 ± 2 ^a,1^	80 ± 1 ^b,1^
UAEP	N.D.	N.D.	N.D.	N.D.	−15.8 ± 0.8 ^b,1^	−15.7 ± 0.1 ^b,1^	113.5 ± 2.5 ^e,2^	211.0 ± 3.5 ^c,1^	82 ± 4 ^a,1^	80 ± 2 ^b,1^
MAE	N.D.	N.D.	N.D.	N.D.	−7.9 ± 0.2 ^c,1^	−7.9 ± 0.3 ^d,1^	200.0 ± 3.1 ^a,2^	275.5 ± 6.7 ^a,1^	64 ± 3 ^b,1^	60 ± 4 ^d,1^
Encapsulate Samples										
ME	176.8	8.5	1.76	2.45	−2.5 ± 0.1 ^b,1^	−1.7 ± 0.1 ^a,2^	128.0 ± 4.0 ^b,2^	156.0 ± 2.2 ^c,1^	61 ± 1 ^a,1^	32 ± 2 ^bc,2^
HAE	150.8	7.9	1.95	2.53	−2.9 ± 0.1 ^a,1^	−1.7 ± 0.1 ^a,2^	133.5 ± 3.4 ^b,2^	157.0 ± 0.8 ^c,1^	56 ± 3 ^b,1^	31 ± 1 ^c,2^
UAEb	223.1	8.0	1.71	2.47	−2.9 ± 0.2 ^a,1^	−1.4 ± 0.1 ^b,2^	113.5 ± 2.0 ^c,2^	154.0 ± 1.2 ^cd,1^	44 ± 1 ^c,1^	37 ± 1 ^a,2^
UAEp	215.0	8.1	1.60	2.55	−2.6 ± 0.2 ^ab,1^	−1.8 ± 0.1 ^a,2^	90.5 ± 0.8 ^d,2^	162.5 ± 1.9 ^b,1^	44 ± 2 ^c,1^	34 ± 1 ^b,2^
MAE	130.0	8.5	2.37	2.53	−2.0 ± 0.1 ^c,1^	1.0 ± 0.1 ^c,2^	128.0 ± 4.0 ^b,2^	156.0 ± 2.2 ^c,1^	61 ± 1 ^a,1^	32 ± 2 ^bc,2^

ME, maceration extraction; HAE, heat-assisted extraction; UAEb, extraction in ultrasound bath; UAEp, extraction by ultrasound probe; MAE, microwave-assisted extraction; d_50_, value of particle size at 50% of cumulative weight; values with the same letter (^a–e^) in each column and number (^1–2^) in each row showed no statistically significant difference (*p* > 0.05; n = 3; analysis of variance, Duncan’s post hoc test); n.d., not determined.

**Table 5:** The transition temperature and enthalpy change of gelatin, freeze-dried and spray-dried wild thyme extracts (HAE and MAE samples) and the corresponding gelatin encapsulates.

Sample	Temperature (°C)		∆H (J/g)	
	FD	SD	FD	SD
Gelatin	80	89.5	137.6	309.7
Extract				
HAE	67.4	74.4	67.1	67.8
MAE	74.3	81.4	68.8	202.5
Encapsulate				
HAE	80.8	86.6	145.1	161.8
MAE	82.8	87.3	122.2	151.0

HAE, heat-assisted extraction; MAE, microwave-assisted extraction.

## Data Availability

The datasets generated during and/or analyzed during the current study are available from the corresponding author on reasonable request.

## Supplementary Materials

Figure S1: Cyclone of spray dryer device after drying of (a) pure wild thyme extract and (b) gelatin-encapsulated extract, Figure S2: Macroscopic view of lyophilized and spray-dried (a) gelatin, (b) wild thyme extract and (c) gelatin-encapsulated wild thyme extract obtained in heat-assisted extraction, Figure S3: Thermograms of lyophilized (I) and spray-dried samples (II) (a) gelatin, wild thyme extracts obtained by (b) high temperature and (c) microwaves, and gelatin-encapsulated extract obtained by (d) high temperature and (e) microwaves; differential scanning calorimetry.

## Sample Availability

Samples of all compounds are available from the authors.
