# Supplementary material for: Freeze vs. Spray Drying for Dry Wild Thyme (Thymus serpyllum L.) Extract Formulations: The Impact of Gelatin as a Coating Material
Source: Molecules. 2021 Jun 28;26(13):3933. doi: 10.3390/molecules26133933 (PMC8271419; doi:10.3390/molecules26133933)
Supplement: Supplementary file 1 [file molecules-26-03933-s001.zip › molecules-1241639-supplementary.pdf]

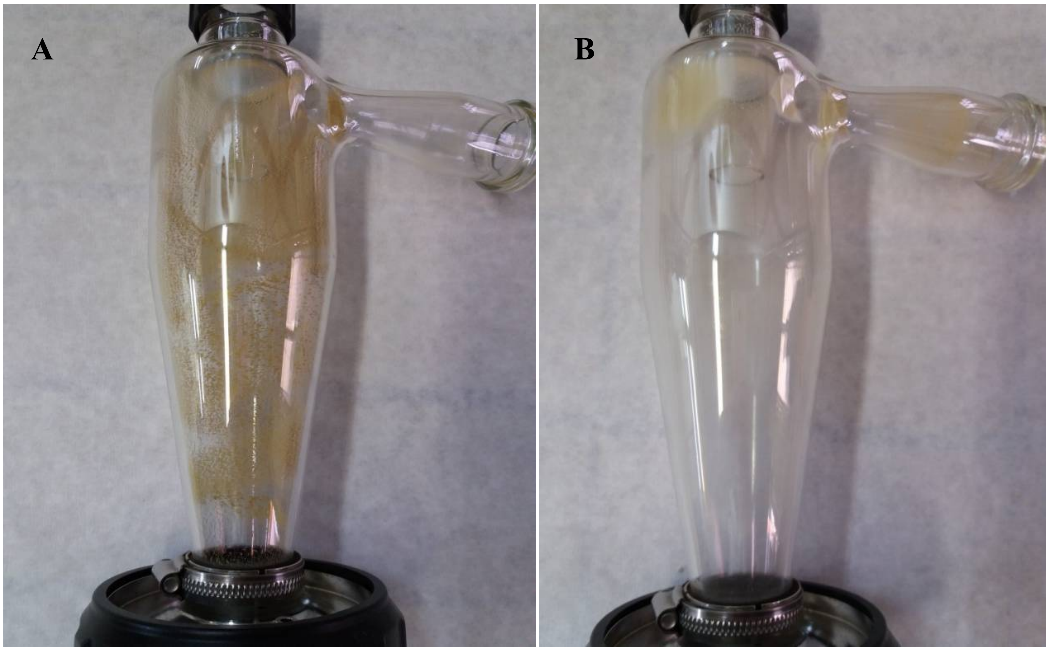

Figure S1. Cyclone of spray dryer device after drying of (A) pure wild thyme extract and (B) gelatin encapsulated extract.

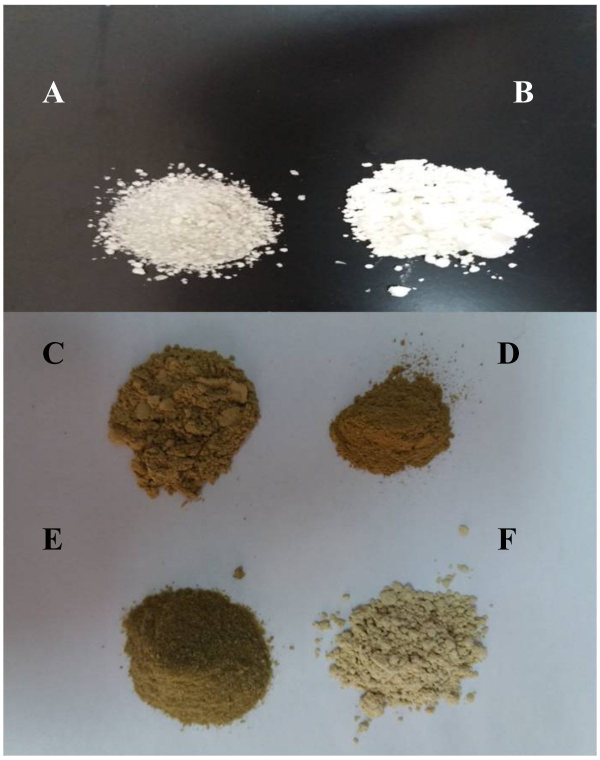

Figure S2. Macroscopic view of lyophilized (A) and spray dried (B) gelatin, lyophilized (C) and spray dried (D)wild thyme extract obtained in heat-assisted extraction and lyophilized (E) and spray dried (F)gelatin encapsulated wild thyme extract.

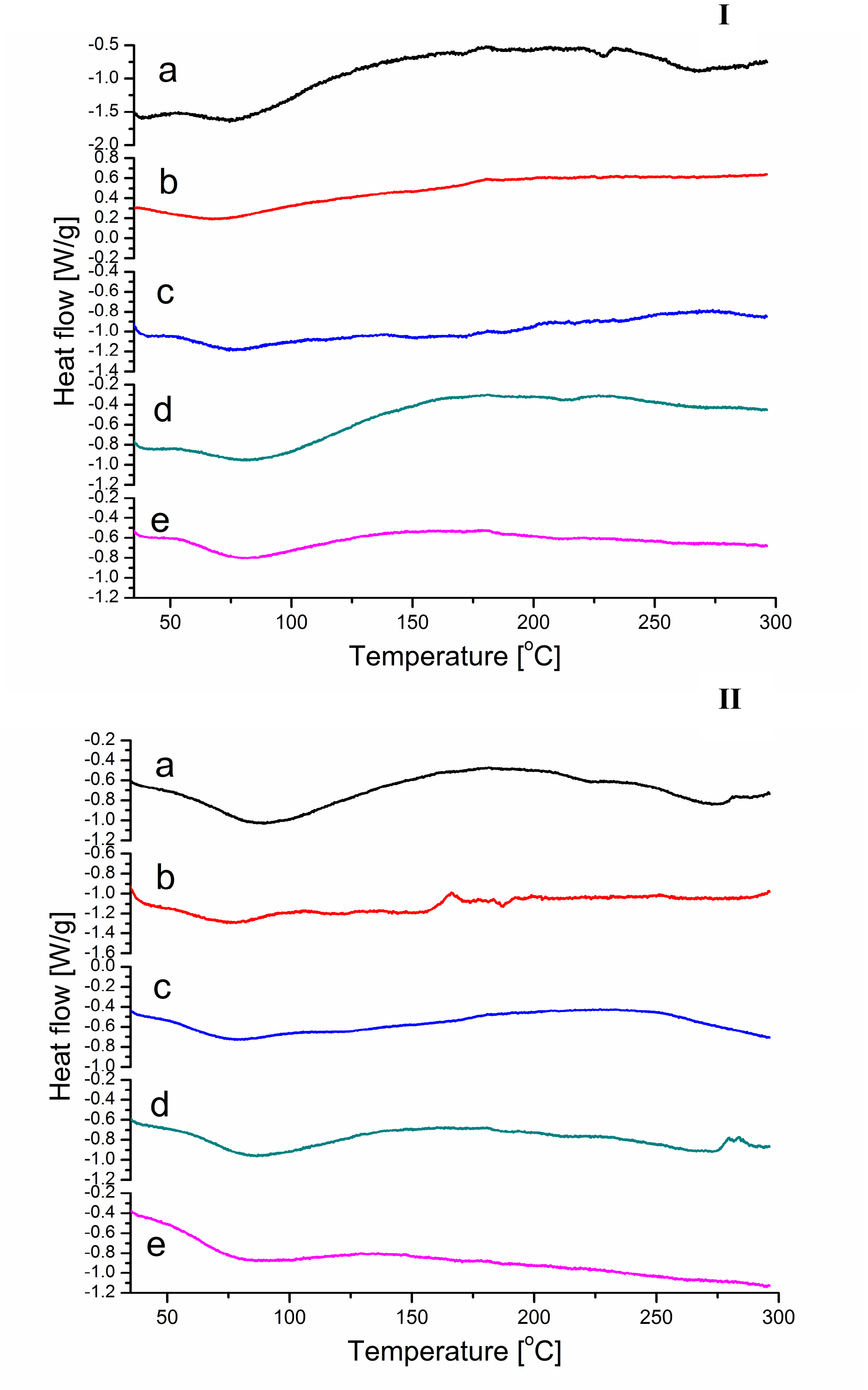

Figure S3. Thermograms of lyophilized (I) and spray dried samples (II) (a) gelatin, wild thyme extracts obtained by (b) high temperature and (c) microwaves, and gelatin encapsulated extract obtained by (d) high temperature and (e) microwaves; differential scanning calorimetry.
